# Anxiety and anxious-depression in Parkinson's disease over a 4-year period: a latent transition analysis

**DOI:** 10.1017/S0033291715002196

**Published:** 2015-11-23

**Authors:** S. Landau, V. Harris, D. J. Burn, J. V. Hindle, C. S. Hurt, M. Samuel, K. C. Wilson, R. G. Brown

**Affiliations:** 1 Department of Biostatistics, Institute of Psychiatry, Psychology and Neuroscience, King's College London, UK; 2 Institute of Neuroscience, Newcastle University, Newcastle upon Tyne, UK; 3 Betsi Cadwaladr University Health Board, Department of Care of the Elderly, Llandudno, UK; 4 University of Bangor, North Wales Organisation for Randomised Trials in Health (NWORTH), UK; 5 School of Health Sciences, City University London, London, UK; 6 Department of Neurology, King's College Hospital, King's Health Partners, London, UK; 7 East Kent Hospitals NHS University Foundation Trust, Ashford, Kent, UK; 8 EMI Academic Unit, University of Liverpool, St Catherine's Hospital, Wirral, UK; 9 Department of Psychology, Institute of Psychiatry, Psychology and Neuroscience, King's College London, UK

**Keywords:** Anxiety, depression, latent transition analysis, Parkinson's disease, subtypes

## Abstract

**Background:**

Depression and anxiety in Parkinson's disease are common and frequently co-morbid, with significant impact on health outcome. Nevertheless, management is complex and often suboptimal. The existence of clinical subtypes would support stratified approaches in both research and treatment.

**Method:**

Five hundred and thirteen patients with Parkinson's disease were assessed annually for up to 4 years. Latent transition analysis (LTA) was used to identify classes that may conform to clinically meaningful subgroups, transitions between those classes over time, and baseline clinical and demographic features that predict common trajectories.

**Results:**

In total, 64.1% of the sample remained in the study at year 4. LTA identified four classes, a ‘Psychologically healthy’ class (approximately 50%), and three classes associated with psychological distress: one with moderate anxiety alone (approximately 20%), and two with moderate levels of depression plus moderate or severe anxiety. Class membership tended to be stable across years, with only about 15% of individuals transitioning between the healthy class and one of the distress classes. Stable distress was predicted by higher baseline depression and psychiatric history and younger age of onset of Parkinson's disease. Those with younger age of onset were also more likely to become distressed over the course of the study.

**Conclusions:**

Psychopathology was characterized by relatively stable anxiety or anxious-depression over the 4-year period. Anxiety, with or without depression, appears to be the prominent psychopathological phenotype in Parkinson's disease suggesting a pressing need to understanding its mechanisms and improve management.

## Introduction

Depression and anxiety are common in Parkinson's disease (PD) (Reijnders *et al.* 2008; Dissanayaka *et al.* 2010), frequently co-occur (Nuti *et al.* 2004; Negre-Pages *et al.* 2010) and are associated with care dependency, poor work and social function, and reduced health-related quality of life (Schrag *et al.* 2000; Riedel *et al.* 2012; Armstrong *et al.* 2014; Chen & Marsh, 2014; Duncan *et al.* 2014). Depression is further associated with faster rate of physical and cognitive decline (Starkstein *et al.* 1992), increased dementia risk (Tandberg *et al.* 1996) and higher mortality (Hughes *et al.* 2004). Despite these facts, good quality evidence for the management of depression is only recently emerging (Dobkin *et al.* 2011; Richard *et al.* 2012) with a recognized paucity of evidence to guide the treatment of anxiety (Seppi *et al.* 2011; Deane *et al.* 2014).

PD is heterogeneous in symptoms and clinical course, suggesting possible pathophysiological disease subtypes and the opportunity to apply stratified treatment approaches to improve outcome (Seppi *et al.* 2011; Berg *et al.* 2013, 2014). We previously reported a comprehensive cross-sectional assessment of mood and mood-related symptoms in a cohort of 513 patients (Prospective Study of Mood State in Parkinson's disease: PROMS-PD;  Brown *et al.* 2011). Latent class analysis (LCA) identified four classes interpreted as: psychologically healthy with low probability of clinically prominent depression and anxiety related symptoms (‘Psychologically healthy’, 60.4%); one characterized by anxiety-related symptoms (‘Anxious’, 22.0%); one with predominantly depressive symptoms (‘Depressed’, 9.0%) and finally a class with both anxiety and depressive symptoms (‘Anxious depressed’, 8.6%). The latter two classes differed on a range of demographic and clinical variables, suggesting that PD depression may be heterogeneous (Brown *et al.* 2011; Burn *et al.* 2012). This paper reports a longitudinal extension of our 2011 study, employing assessment over a 4-year period to permit a more reliable assessment of mood-related subtypes; characterize change in depressive and anxious symptomatology as the disease progresses, and examine associated clinical and demographic features.

## Method

### Longitudinal PD cohort

PROMS-PD (UKCRN ID 2519) recruited participants over a 14-month period from specialist PD or movement disorder outpatient clinics across the UK (Brown *et al.* 2011). Eligibility criteria at inclusion were a clinical diagnosis of idiopathic PD, the ability to provide informed consent at entry and living within travelling distance from a study centre. Entry exclusion criteria were sensory loss or communication difficulty sufficient to interfere with assessment. After baseline assessment (year 1) participants were re-contacted 6-monthly and assessed annually. Drop-out at each contact was classified as death; not-assessable or withdrawal from the study due to worsening of physical or mental health or cognitive impairment; withdrawal for other reasons; loss of contact or moved out of area, and change in primary diagnosis.

### Baseline clinical and demographic measures

Participants were assessed as described in full elsewhere (Brown *et al.* 2011; Burn *et al.* 2012). Demographic information included gender, current age and socio-economic status (Office for National Statistics, 2000). Disease-related variables included age of PD onset, side of PD symptom onset and current antiparkinsonian medication from which a l-dopa equivalent daily dose (LEDD) was calculated (Tomlinson *et al.* 2010). Motor disability, symptom severity and motor complications were assessed using the Unified Parkinson's Disease Rating Scale (UPDRS; Fahn *et al.* 1987) parts II-IV, plus the Hoehn and Yahr Scale (Hoehn & Yahr, 1967), and Schwab and England Scale (Schwab & England, 1969). Rate of progression of motor symptoms at baseline (Lewis *et al.* 2005) and motor phenotype [Postural Instability and Gait (PIGD) or Tremor-Dominant/Indeterminate] were derived from the UPDRS (Jankovic *et al.* 1990). Cognition was assessed using the Addenbrooke's Cognitive Examination – Revised (ACE-R; Mioshi *et al.* 2006), an extended mental status examination assessing a range of neuropsychological processes relevant to PD. Burden of health complaint was assessed using the Duke University Older Americans Resources and Services (OARS) physical health measure (Whitelaw & Liang, 1991), including a checklist of physical health complaints common in older adults.

### Assessment of depressive- and anxiety-related symptoms

**Fig. 1.**
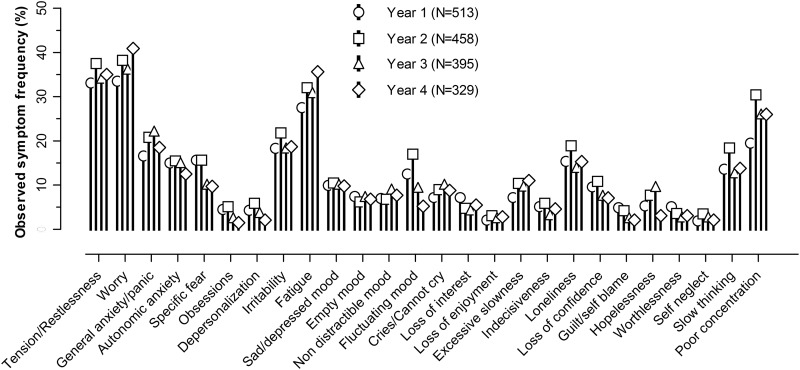
Observed Geriatric Mental State prominent symptom frequencies by year.

Each year, depressive, anxiety and related symptoms were assessed using a semi-structured interview based on the Geriatric Mental State (GMS; Copeland *et al.* 1976). Symptoms were rated and then recoded as ‘prominent’ (1) or ‘absent/normal’ or ‘present but not prominent’ (0). Analyses were restricted to 26 GMS items reported as prominent in at least 2% of the sample at baseline (see Fig. 1). Depression severity was assessed with the Hamilton Depression Rating Scale (HAMD; Hamilton, 1960). The Hospital Anxiety and Depression Scale (HADS) was used for self-report (Zigmond & Snaith, 1983). A history of depressive or anxiety disorder was ascertained by interview.

### Latent transition analyses (LTAs)

LTA is an extension of LCA which assumes that there exist unobserved (latent) categorical variables (classes) that explain the associations between observed measures. Two sets of parameters are estimated: (i) probabilities describing the marginal distribution of the latent classes at baseline and transition probabilities between latent classes over time and (ii) conditional probabilities, i.e. probabilities that a symptom is present given the participant belongs to a class. LTAs (Graham *et al.* 1991; Collins & Lanza, 2010) were carried out to identify depression and anxiety related subtypes in PD, characterize their profiles and describe change over time (transitions). Our LTA approach is similar to that used elsewhere (Lamers *et al.* 2012). Specifically, we fit LTA models to a matrix of 513 patients on 26 binary GMS symptom scores at each of four annual time-points. We refer to the probabilities across the GMS items as the *class profiles* and use them for interpretation and labelling.

We assume that class profiles themselves do not vary over time, although we tested this assumption empirically by comparing the fit to that of a model that allowed all the conditional probabilities to vary (Collins & Lanza, 2010). As the LTAs carried out here were extensions of an LCA performed on the cross-sectional baseline data (Brown *et al.* 2011), we planned to fit a four-class model, but also re-assessed the number of classes empirically using several model fit indices.

We wished to employ all the available GMS data in the LTA modelling but without relying on the restrictive assumption that GMS scores were missing completely at random (MCAR). Instead we used survival analysis to identify baseline variables that predicted drop-out from the study and then extended the LTA model to include these variables by allowing them to predict class membership at each time point. The derived parameter estimates are valid under a less restrictive missing at random (MAR) assumption. Drop-out was measured every 6-month interval with time intervals frequently being right-censored. Complementary log-log regression was used to model the effect of a baseline variable on the hazard of dropping out in the next 6-month interval given that a participant is still in the study (Prentice & Gloeckler, 1978). The approach relies on a proportional hazards assumption. The effect of each putative baseline predictor of drop-out was assessed separately resulting in a largish list of empirically identified predictors. A smaller representative predictor subset was identified for inclusion in the LTA choosing individual predictors that represented subsets of correlated predictors.

Stepwise multinomial logistic regression was used to assess whether baseline variables could predict path memberships of interest. A list of putative baseline predictors representing different constructs was used and a best prediction model established by forward variable selection. The forward variable selection was carried out by initially fitting multinomial logistic regression models with each predictor as a single covariate and selecting the best predictor by comparing the *χ*^2^ goodness-of-fit statistic. At each subsequent stage the effect of adding each of the remaining predictors to the model was assessed using a likelihood ratio test and the next best predictor selected on the basis of statistical significance. The procedure was terminated when the likelihood ratio test was no longer significant for any of the remaining predictors.

Statistical analyses were carried out in Stata v. 11 (StataCorp, 2009) and Mplus v. 7 (Muthen & Muthen, 2007).

## Ethical standards

The authors assert that all procedures contributing to this work comply with the ethical standards of the relevant national and institutional committees on human experimentation and with the Helsinki Declaration of 1975, as revised in 2008. (Ethics reference 07/MRE01/9).

## Results

### Sample characteristics and longitudinal GMS assessments

Of 941 patients invited to participate, 525 consented but 12 withdrew before baseline assessment. The baseline sample (*N* = 513) has been described previously (Brown *et al.* 2011; Burn *et al.* 2012). Briefly, there was a wide range of PD duration and severity, although a majority were Hoehn and Yahr stages II–III with a non-tremor dominant motor profile. Almost all (95%) were taking antiparkinsonian drugs. About a third of the sample showed evidence of cognitive impairment. Analysable data were available from 458 (89.2%) at year 2, 395 (77.0%) at year 3 and 329 (64.1%) at year 4 (see Supplementary Fig. S1). There were 44 (8.6%) deaths; 45 (8.8%) became unassessable or withdrew from the study on health grounds; 48 (9.4%) withdrew for other reasons (e.g. too busy, no longer interested or no reason given), while 42 (8.2%) moved away or were uncontactable. Ten (1.9%) were re-diagnosed with either essential tremor (*N* = 3), progressive supranuclear palsy (*N* = 1), multiple system atrophy (*N* = 4) or had undergone a negative dopamine transporter scan (*N* = 2). As these numbers were small and all participants met diagnostic criteria at inclusion, they were retained in the analysis to better reflect the reality of the clinical setting.

The observed characteristics of the remaining sample at each time point were not subject to formal statistical comparison, and interpretation is subject to the effects of non-random drop-out. Nevertheless, the data indicated disease progression as evidenced by worsening of observed mean UPDRS scores, Hoehn and Yahr stages and increasing LEDD (see Supplementary Table S1). The overall rate of observed depression (HAMD ⩾10) increased from 20.5% at baseline to 24.1% of those remaining in the study at year 4, while use of antidepressant/anxiolytic medication increased from 23.5% to 30.4% over the same period. Fig. 1 shows the profile of observed frequencies of prominent GMS symptoms at each assessment point. Consistently over time the most common symptoms were worry, subjective tension and restlessness and fatigue with an observed prevalence of ⩾30% followed by poor concentration, irritability, symptoms of general anxiety and panic and loneliness. Other symptoms were observed in ⩽20% of the total sample across the 4 years.

### LTA-defined PD subtypes

Table 1 shows fit indices for different numbers of classes based on the binary GMS scores alone. Akaike's Information Criterion (AIC) and sample size adjusted Bayesian Information Criterion (BIC) indicate the best fit for six classes, while the BIC and entropy support a four-class solution. We selected the latter as the most interpretable, for consistency with our previous LCA solution and to avoid small classes containing <5% of participants.

**Table 1.** Information criteria and entropies for fitted LTA models with different numbers of classes

No. of classes	AIC	BIC	Sample size adjusted BIC	Entropy
2	24 896	25 146	24 959	0.867
3	24 215	24 631	24 320	0.829
4	23 892	24 499	24 045	0.836
5	23 711	24 534	23 918	0.809
6	23 575	24 640	23 843	0.830

LTA, Latent transition analysis; AIC, Akaike's Information Criterion; BIC, Bayesian Information Criterion.

For the first three indices (AIC, BIC, sample size adjusted BIC) a lower value indicates a better model fit. Entropy measures how well participants are classified with higher values indicating better classification.

Model fit indices for a model assuming full parameter invariance (AIC 22 834, BIC 23 736, sample size adjusted BIC 23 053) were better than those of a model that allowed class profiles to vary over time (AIC 23 601, BIC 25 271, sample size adjusted BIC 23 599). Thus, we continued to assume that class profiles did not vary and use the estimated conditional probabilities to summarize depressive and anxiety symptoms within classes.

The following baseline variables were associated with earlier drop-out (hazard ratios that differed from 1 (*p* < 0.05) in the complementary log-log regressions): older age, later age of PD onset, longer PD duration, higher UPDRS-III score, higher HAMD score, higher HADS anxiety and depression scores, lower ACE-R score, worse Schwab and England score, and Hoehn and Yahr stages IV or V. To reflect these correlated concepts in the LTA models, age at baseline, duration of PD, UPDRS-III score, HADS anxiety and depression and ACE-R score were selected for inclusion.

**Fig. 2.**
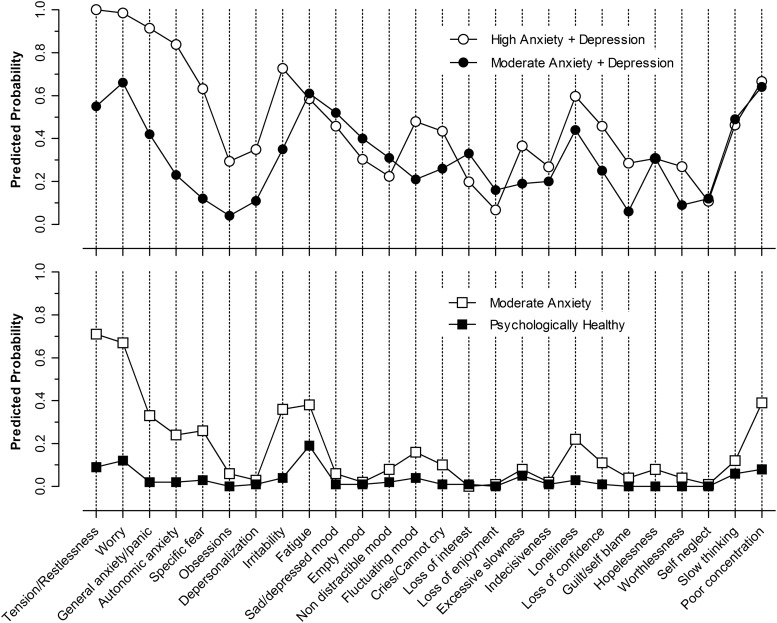
Estimated profiles of depression- and anxiety-related Parkinson's disease latent transition analysis classes.

Interpretation of the LTA model was based on examination of the symptom profiles for the four latent classes (see Fig. 2). The first class (open circles) represents a high predicted probability (0.60–1.00) of anxiety symptoms, particularly subjective tension and worry, symptoms of generalized anxiety/panic, autonomic symptoms of anxiety, irritability and poor concentration. Other common symptoms (probability 0.40–0.59) included fatigue, depressed mood, crying, indecisiveness and slowed thinking. This class is labelled ‘High anxiety + depression’. Class 2 (solid circles), shows a similar profile but with lower probabilities of the main anxiety-related symptoms and is labelled ‘Moderate anxiety + depression’. Class 3 (open squares) is characterized mainly by moderate probabilities of anxiety symptoms without evident depression and is labelled ‘Moderate anxiety’. Finally, class 4 (solid squares) was characterized by low probability of any prominent symptoms and labelled ‘Psychologically healthy’.

The four-class LCA solution derived using the entire 4 years varies somewhat from our previous LTA solution from the baseline data alone (Brown *et al.* 2011). A cross-tabulation of the classifications at baseline revealed that the solutions matched for 87% of the sample with high correspondence (93%) for the ‘Psychologically healthy’ in the two analyses. Similarly 87% of those allocated to the LCA ‘Anxiety’ class were allocated to the LTA ‘Moderate anxiety’ class. For the two smaller classes, 59% of those previously labelled by LCA ‘Anxious depressed’ were now allocated to the ‘High anxiety + depression’ LTA class and 38% to ‘Moderate anxiety + depression’. Similarly, 64% of those in the original LCA class ‘Depressed’ ended up in the new ‘Moderate anxiety + depression’ LTA class, while 23% were labelled ‘Moderate anxiety’.

Because of the association between higher levels of depression and anxiety at baseline and subsequent loss to follow-up we would expect different drop-out rates across the four classes. However, the inclusion of predictors of drop-out in the LTA model means that such differential drop-out would not affect the analysis or interpretation of the results.

### Transitions between PD subtypes and trajectory paths

**Fig. 3.**
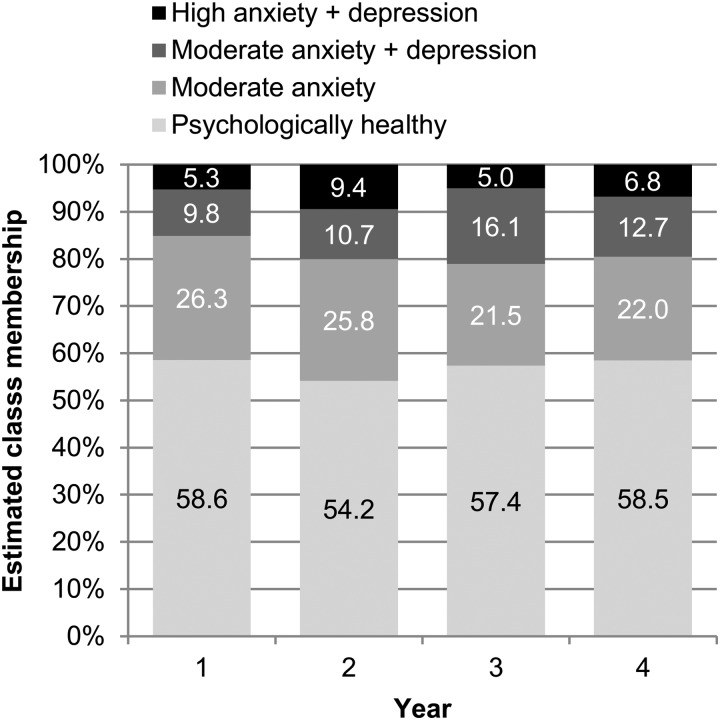
Estimated proportions (%) of latent transition analysis class membership at each time-point.

Fig. 3 shows the estimated marginal proportions of the four classes at each time-point. The ‘Psychologically healthy’ class was most common, followed by the ‘Moderate anxiety’ class, and two anxious and depressed classes. There was a trend towards an increase in the combined depression-related classes, from 15.1% at baseline to 19.5–21.1% in subsequent years, while the ‘Moderate anxiety’ class showed a corresponding decrease.

**Fig. 4.**
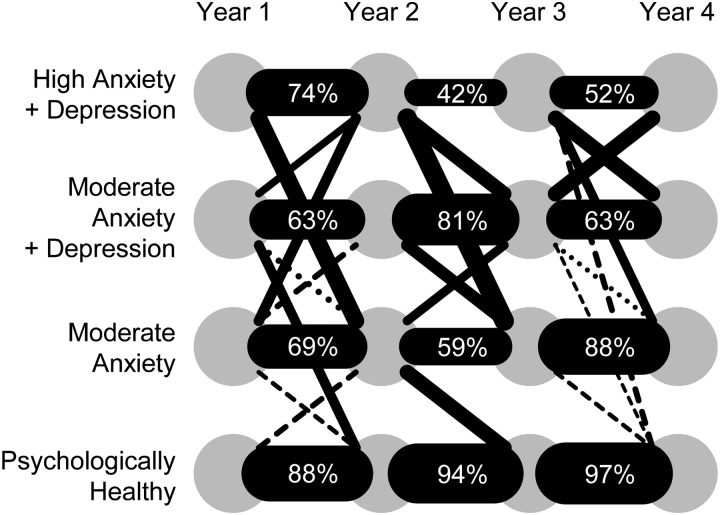
Estimated frequencies of class transitions. The thickness of each black connecting bar (or dashed line) is proportional to the estimated transition frequency. Numerical frequencies are shown where >40%. Dashed lines indicate frequencies of 5–10%. For clarity, class transition frequencies of <5% are not shown (see Supplementary Table S3 for full numerical data).

We used the fitted LTA model to classify every patient at each time-point according to their maximum posterior estimated class probability. Observed trajectories are represented graphically in Fig. 4 (see also Supplementary Table S3). The most common paths were those with membership of the same class over adjacent years. Overall, an estimated 51.1% [95% confidence interval (CI) 46.7–55.6] remained in the ‘Psychologically healthy’ class throughout the 4 years (which we call the ‘Remaining healthy’ path) while 33.7% (95% CI 29.5–38.0) stayed in one or other of the remaining classes (‘Remaining distressed’ path). Only 8.6% (95% CI 6.1–11.1) initially healthy were reclassified into one of the other classes (‘Becoming distressed’ path), while 6.5% (95% CI 4.3–9.7), of those initially ‘distressed’ were later reclassified as ‘Psychologically healthy’ (‘Becoming well’ path).

### Baseline predictors of trajectory paths

What predicts membership of these paths? Because we were modelling longitudinal patterns of change in the context of a chronic and progressive condition we limited the analysis to the ‘Remaining distressed’ and ‘Becoming distressed’ paths with ‘Remaining healthy’ as the reference path. Additionally, it was assumed that ‘Becoming well’ might reflect successful management of the depression and/or anxiety rather than reflecting particular baseline characteristics.

The following list of baseline demographic and clinical variables were considered a priori as potential predictors of trajectory paths: gender, age, age of onset of PD, LEDD, UPDRS-III, PD motor phenotype, estimated rate of progression of motor symptoms, presence of motor fluctuations, depression severity (HAMD), cognitive function (ACE-R), burden of physical health conditions (OARS), socio-economic status (NS-SEC) and history of psychiatric illness. The final model included HAMD score, age of PD onset and history of past mental illness as predictors of trajectory paths [model fit likelihood ratio *χ*^2^(6) = 194.33, *p* < 0.0001, pseudo-*r*^2^ = 0.238]. Younger age of onset predicted being on the ‘Becoming distressed’ path relative to ‘Remaining healthy’ [odds ratio (OR) per extra year = 0.95, 95% CI 0.93–0.98, *z* = −3.29, *p* < 0.001] (i.e. for every year older at PD onset there was a 5% lower risk of becoming distressed having been psychologically healthy initially). ‘Remaining distressed’ was predicted by higher baseline HAMD (OR per extra point on scale = 1.35, 95% CI 1.27–1.44, *z* = 9.28, *p* < 0.001), younger age of PD onset (OR 0.97, 95% CI 0.95–0.99, *z* = −2.75, *p* = 0.01) and history of past psychiatric illness (OR 2.17, 95% CI 1.23–3.83, *z* = 2.68, *p* = 0.01).

It should be noted that this analysis is limited to baseline predictors of broadly defined paths. We did not consider the large number of potential individual transitions, nor factors that may have changed in the preceding 12 months before each assessment. Thus changes in medication and health status, life events, etc. may have additionally explained some of the individual transitions observed.

## Discussion

We consider here the present findings in relation to our previous cross-sectional analysis and subtype classification (Brown *et al.* 2011) before discussing the results in the context of other longitudinal studies of depression and anxiety in PD. We then consider the implications for our conceptualization of depression and anxiety, its clinical management and directions for future research.

The LTA enhances our previous work investigating latent classes at a single time point (Brown *et al.* 2011) as it makes use of all available data over an extended period of cohort observation incorporating baseline predictors of missingness. The profiles of the four-class solutions from the present LTAs were broadly similar to those from the previous, but more restricted, LCA with close agreement in class membership. They differed in that the ‘Depressed’ class profile was better characterized as ‘Moderate anxiety + depression’. This differed from the ‘High anxiety + depression’ class profile mainly in the prominence of anxiety symptoms (Fig. 2). The existence of two anxious-depressed classes (comprising almost 20% of our sample by year 4) and a further 20% anxious patients (class 3), points strongly to anxiety being the most prevalent and prominent mood-related non-motor feature of PD (Fig. 3), co-occurring 50% of the time with depressive symptoms.

It is notable that the rates of anxiety-related symptom profiles over the years (40–45%) was higher than the prevalence of anxiety indicated by the HADS (Anxiety score ⩾11) of 20–25% (Supplementary Table S2). This may reflect differences in the nature of the assessment (clinician rated using semi-structured interview for the GMS and self-report for the HADS) or a lower sensitivity to detect clinically meaningful symptomatology from the HADS. However, the results indicate that evidence derived from the HADS, and potentially other self-report scales, may underestimate the burden of prominent anxiety-related symptomatology in PD.

The close association between depression and anxiety observed here is consistent with previous findings that two-thirds of PD patients with a depressive disorder had a co-morbid anxiety disorder (Menza *et al.* 1993), and that the presence of depression was associated with a five-fold increased risk of co-morbid anxiety (Qureshi *et al.* 2012). Importantly, our current data-driven methods failed to identify a class with a profile characterized by predominantly depressive symptoms. As such, the present results do not support the existence of depression subtypes with potentially different aetiologies and mechanisms and so requiring different treatments. Rather, the empirically defined PD subgroups are better characterized by the severity of anxiety symptoms and the presence or absence of co-occurring depression. However, the lack of a non-anxious depressed class in the LTA does not imply that depression without anxiety is absent in PD. The LTA classes are defined on the basis of a profile of symptom probabilities and do not indicate the absolute presence of absence of a symptom. Additionally, constraining the model to four classes may have masked smaller groupings that would have emerged if the model had allowed for a larger number of classes.

The validity of this LTA depends upon the assumption that the data are missing at random, i.e. that the probability of being missing is dependent only on variables which are incorporated by the model. This assumption is reasonable as the additional variables included as predictors of latent classes capture reasons for drop-out, in particular worsening health or death. Other reasons for drop-out, such as having moved out of the area, may be viewed as chance occurrences. It is acknowledged that additional factors influencing drop-out may have been missed, which would introduced bias. We have assumed that any such bias will be relatively small and as such will be more than compensated for by the gains made from using all the available data.

Although not the primary aim, the present report provides novel prospective data on the natural history of depression and anxiety in a large, well characterized cohort. The observed symptoms showed a remarkably consistent profile over the four time-points (Fig. 1). There was relatively little change in individual symptom prevalence, and observed overall mean depression and anxiety severity as indicated by the mean HADS and HAMD scores remained relatively stable (see Supplementary Table S1). However categorically defined depression (HAMD ⩾10) did increase slightly from 20.5% of the sample at baseline to 24.1% of those remaining in the study after 4 years. In one of the few previously published longitudinal studies (DATATOP), 795 newly diagnosed PD patients were followed for up to 7 years, by which time an estimated 24% scored ⩾10 on the HAMD (Vu *et al.* 2012). Depression was also relatively stable in an earlier 12-month longitudinal study (Starkstein *et al.* 1992).

An important contribution of this study is the characterization of change over time in terms of transitions between mood-related classes. Examination of the data and analyses revealed a predominant tendency for stability rather than change (Fig. 4 and Supplementary Table S3). In particular over 50% of the cohort remained in the psychologically healthy class over the entire 4 years of study, despite facing the likely challenge of a progressive condition and prospect of future worsening. A proportion (13.7% in year 1, to 23.5% in year 4) were taking antidepressant/anxiolytics and so, for some, psychological health may indicate successful treatment outcome. For the remainder, however, it is likely to reflect other factors. Resilience is a multidimensional construct that allows individuals to navigate and respond to the challenges of a chronic stressor such as disease to protect life-satisfaction and well-being. Social and lifestyle factors such as social networks and physical activity may play a role, while adaptive illness beliefs and coping style can play an important role in buffering the effects of disease, either the greater active use of problem-orientated coping, or lesser reliance on strategies involving avoidance or emotion (Evans & Norman, 2009; Hurt *et al.* 2011, 2012). Complex biologically and environmentally determined dispositional characteristics (traits) may also play a protective role including optimism (Hurt *et al.* 2013; Gison *et al.* 2014) or a lifelong low tendency to engage in worry or rumination in response to stressors and threats (Michl *et al.* 2013). At a more fundamental level there is growing interest in how low biological sensitivity of the hypothalamic-pituitary-adrenal axis and sympathetic nervous system to stressors may be protective, and amenable to behavioural interventions such as physical exercise (Silverman & Deuster, 2014).

Just as psychologically healthy individuals tended to remain so over the course of the 4 years, we showed that those with prominent symptoms of anxiety and/or depression at baseline tended to remain psychologically distressed. Antidepressant/anxiolytic use, although higher than in the psychologically healthy class, was still less than might be expected (see Supplementary Table S3) suggesting possible under-recognition or under-treatment by clinicians as reported elsewhere (Shulman *et al.* 2002; Weintraub *et al.* 2003), or patient reluctance to take more medication (Dobkin *et al.* 2013). Prolonged distress might also indicate the presence of vulnerability factors opposite to those associated with resilience. Although not a comprehensive assessment, our analysis identified only younger age of onset, higher baseline depression levels and a prior history of depressive illness as the main predictors of persistent psychological morbidity relative to continued health. Other demographic and PD-related factors such as higher LEDD, the presence of motor fluctuations, cognitive impairment and motor phenotype were not predictive once the effect of the above three variables was accounted for. Few participants became distressed, having been well initially and only younger age of onset predicted this trajectory. However, this reinforces the vulnerability of this group and the need for clinical vigilance.

Consistent with evidence that anxiety increases the risk of depression onset or relapse in older adults (Andreescu *et al.* 2007) 25.7% (*n* = 29) of those in our ‘Moderate anxiety’ class in year 1 transitioned into one of the two anxiety + depression classes in year 2 (Supplementary Table S3). At the same time 36.1% (*n* = 31) transitioned in the opposite direction, suggesting a remission of depression but with significant residual anxiety.

It should be noted that the reported evidence on stability and transitions needs to be interpreted in the context of the 12-month assessment intervals. This means that shorter term transitions (e.g. from psychologically healthy to distressed and back to healthy) would not have been detected.

What are the implications of these findings for treatment and the design of clinical trials? Good quality evidence for the efficacy of pharmacotherapy for depression remains limited, inconsistent and indicates only low to moderate effect sizes (Weintraub *et al.* 2005; Menza *et al.* 2009). In the largest trial to date (SAD-PD), significant effects were observed for mean depression severity change at 12 weeks treatment with paroxetine and venlafaxine XR. However, response and remission rates did not differ from placebo (Richard *et al.* 2012). However a third or more of those receiving active treatment failed to show a clinical response (⩾50% decrease in HAM-D) (32% paroxetine, 47% venlafaxine), while the majority failed to remit (defined as HAMD ⩽7) (56% paroxetine and 63% venlafaxine). These results are consistent with other depression RCTs in older adults. Low remission and response rates are typical and associated with factors including co-morbid physical health conditions and cognitive change, particularly executive dysfunction (Alexopoulos *et al.* 2002), both very relevant to PD. A further predictor of poor treatment outcome is co-morbid anxiety. In the SAD-PD trial, high anxiety predicted worse antidepressant response (Moonen *et al.* 2014), a finding shown in RCTs of non-PD depression (Fava *et al.* 2008; Penninx *et al.* 2011) including in late-life depression (Andreescu *et al.* 2007; Cohen *et al.* 2009). Relapse rates tend to be high in such cases, perhaps because of a larger burden of side effects and lower compliance with maintenance therapy. While future antidepressant trials might seek to exclude patients with significant anxiety to evaluate their effectiveness in ‘pure’ depression, such evidence may be of limited value to guide the treatment of depression in PD where current evidence suggests that anxiety is such a prominent accompaniment.

Clinically, we need to be vigilant not just for the presence of depression, but the symptoms of anxiety and associated risk factors. As well as being a source of distress and disability, evident anxiety may indicate the possible presence of unrecognized depression, an increased risk of future depression and poor treatment outcome. For research, we need a far better understanding of the factors associated with onset, maintenance and outcome of anxiety if it is a characteristic component of the mood phenotypes in PD. Currently, our understanding of anxiety and its treatment lags far behind that of depression (Seppi *et al.* 2011), with no clinical trials to date with anxiety as the target and none in progress (ClinicalTrials.gov). This evidential gap was powerfully illustrated in the results of a recent systematic exercise to identify current gaps in clinical management commissioned by Parkinson's UK (Deane *et al.* 2014). Involving patients, carers, family members and health and social care professionals, it ranked ‘reducing stress and anxiety’ second in terms of evidential uncertainty below the management of balance and falls and above a wide range of other motor and non-motor symptoms. What form such treatment might take remains unclear without the underpinning research, but might best encompass a broad based, stepped-care approach with a combination of targeted psychological and pharmacological interventions.

## Conclusions

The present study highlights the prevalence and potential clinical significance of anxiety-related symptoms in PD psychopathology, either in relative isolation or in the presence of depressive symptoms. A high level of stability is observed over time, both good psychological health pointing to resilience in some patients, but also in sustained psychological distress in many others extending over periods of years despite the best efforts of healthcare professionals. The need for better management of anxiety symptoms is becoming recognized and may be key to improving patient outcome. Primary trials of pharmacological and psychological treatments for anxiety are urgently needed, in addition to research into risk, aetiological and maintaining factors.

## Supplementary material

For supplementary material accompanying this paper visit http://dx.doi.org/10.1017/S0033291715002196. click here to view supplementary material

## References

[ref1] Alexopoulos GS, Buckwalter K, Olin J, Martinez R, Wainscott C, Krishnan KR (2002). Comorbidity of late life depression: an opportunity for research on mechanisms and treatment. Biological Psychiatry 52, 543–558. 12361668 10.1016/s0006-3223(02)01468-3

[ref2] Andreescu C, Lenze EJ, Dew MA, Begley AE, Mulsant BH, Dombrovski AY, Pollock BG, Stack J, Miller MD, Reynolds CF (2007). Effect of comorbid anxiety on treatment response and relapse risk in late-life depression: controlled study. British Journal of Psychiatry 190, 344–349. 17401042 10.1192/bjp.bp.106.027169

[ref3] Armstrong MJ, Gruber-Baldini AL, Reich SG, Fishman PS, Lachner C, Shulman LM (2014). Which features of Parkinson's disease predict earlier exit from the workforce? Parkinsonism and Related Disorders 20, 1257–1259. 25179494 10.1016/j.parkreldis.2014.08.005

[ref4] Berg D, Lang AE, Postuma RB, Maetzler W, Deuschl G, Gasser T, Siderowf A, Schapira AH, Oertel W, Obeso JA, Olanow CW, Poewe W, Stern M (2013). Changing the research criteria for the diagnosis of Parkinson's disease: obstacles and opportunities. Lancet Neurology 12, 514–524. 23582175 10.1016/S1474-4422(13)70047-4

[ref5] Berg D, Postuma RB, Bloem B, Chan P, Dubois B, Gasser T, Goetz CG, Halliday GM, Hardy J, Lang AE, Litvan I, Marek K, Obeso J, Oertel W, Olanow CW, Poewe W, Stern M, Deuschl G (2014). Time to redefine PD? Introductory statement of the MDS Task Force on the definition of Parkinson's disease. Movement Disorders 29, 454–462. 24619848 10.1002/mds.25844 PMC4204150

[ref6] Brown RG, Landau S, Hindle JV, Playfer J, Samuel M, Wilson KC, Hurt CS, Anderson RJ, Carnell J, Dickinson L, Gibson G, van SR, Sellwood K, Thomas BA, Burn DJ (2011). Depression and anxiety related subtypes in Parkinson's disease. Journal of Neurology Neurosurgery and Psychiatry 82, 803–809. 10.1136/jnnp.2010.213652 21217155

[ref7] Burn DJ, Landau S, Hindle JV, Samuel M, Wilson KC, Hurt CS, Brown RG (2012). Parkinson's disease motor subtypes and mood. Movement Disorders 27, 379–386. 22162098 10.1002/mds.24041

[ref8] Chen JJ, Marsh L (2014). Anxiety in Parkinson's disease: identification and management. Therapeutic Advances in Neurological Disorders 7, 52–59. 24409202 10.1177/1756285613495723 PMC3886380

[ref9] Cohen A, Gilman SE, Houck PR, Szanto K, Reynolds CF III (2009). Socioeconomic status and anxiety as predictors of antidepressant treatment response and suicidal ideation in older adults. Social Psychiatry and Psychiatric Epidemiology 44, 272–277. 18818858 10.1007/s00127-008-0436-8 PMC2662042

[ref10] Collins LM, Lanza ST (2010). Latent Class and Latent Transition Analysis with Applications in the Social, Behavioral and Health Sciences. John Wiley & Sons, Inc.: Hoboken, NJ.

[ref11] Copeland JR, Kelleher MJ, Kellett JM, Gourlay AJ, Gurland BJ, Fleiss JL, Sharpe L (1976). A semi-structured clinical interview for the assessment of diagnosis and mental state in the elderly: the Geriatric Mental State Schedule. I. Development and reliability. Psychological Medicine 6, 439–449. 996204 10.1017/s0033291700015889

[ref12] Deane KH, Flaherty H, Daley DJ, Pascoe R, Penhale B, Clarke CE, Sackley C, Storey S (2014). Priority setting partnership to identify the top 10 research priorities for the management of Parkinson's disease. BMJ Open 4, . 10.1136/bmjopen-2014-006434 PMC4281559 25500772

[ref13] Dissanayaka NN, Sellbach A, Matheson S, O'Sullivan JD, Silburn PA, Byrne GJ, Marsh R, Mellick GD (2010). Anxiety disorders in Parkinson's disease: prevalence and risk factors. Movement Disorders 25, 838–845. 20461800 10.1002/mds.22833

[ref14] Dobkin RD, Menza M, Allen LA, Gara MA, Mark MH, Tiu J, Bienfait KL, Friedman J (2011). Cognitive-behavioral therapy for depression in Parkinson's disease: a randomized, controlled trial. American Journal of Psychiatry 168, 1066–1074. 21676990 10.1176/appi.ajp.2011.10111669 PMC3186855

[ref15] Dobkin RD, Rubino JT, Friedman J, Allen LA, Gara MA, Menza M (2013). Barriers to mental health care utilization in Parkinson's disease. Journal of Geriatric Psychiatry and Neurology 26, 105–116. 23589410 10.1177/0891988713481269 PMC3644337

[ref16] Duncan GW, Khoo TK, Yarnall AJ, O'Brien JT, Coleman SY, Brooks DJ, Barker RA, Burn DJ (2014). Health-related quality of life in early Parkinson's disease: the impact of nonmotor symptoms. Movement Disorders 29, 195–202. 24123307 10.1002/mds.25664

[ref17] Evans D, Norman P (2009). Illness representations, coping and psychological adjustment to Parkinson's disease. Psychology and Health 24, 1181–1196. 20204987 10.1080/08870440802398188

[ref18] Fahn S, Elton RL, members of the UPDRS development committee (1987). Unified Parkinson's Disease Rating Scale In Recent Developments in Parkinson's Disease (ed. S. Fahn , C. D. Marsden and D. B. Calne ), pp. 153–164. Macmillan Health Care Information: Florham Park, NJ.

[ref19] Fava M, Rush AJ, Alpert JE, Balasubramani GK, Wisniewski SR, Carmin CN, Biggs MM, Zisook S, Leuchter A, Howland R, Warden D, Trivedi MH (2008). Difference in treatment outcome in outpatients with anxious versus nonanxious depression: a STAR*D report. American Journal of Psychiatry 165, 342–351. 18172020 10.1176/appi.ajp.2007.06111868

[ref20] Gison A, Dall'Armi V, Donati V, Rizza F, Giaquinto S Irccs San Raffaele Pisana Rome Italy (2014). Dispositional optimism, depression, disability and quality of life in Parkinson's disease. Functional Neurology 29, 113–119. 25306121 PMC4198159

[ref21] Graham JW, Collins LM, Wugalter SE, Chung NK, Hansen WB (1991). Modeling transitions in latent stage-sequential processes: a substance use prevention example. Journal of Consulting and Clinical Psychology 59, 48–57. 2002142 10.1037//0022-006x.59.1.48

[ref22] Hamilton M (1960). A rating scale for depression. Journal of Neurology Neurosurgery and Psychiatry 23, 56–62. 10.1136/jnnp.23.1.56 PMC495331 14399272

[ref23] Hoehn MM, Yahr MD (1967). Parkinsonism: onset progression and mortality. Neurology 17, 427–442. 6067254 10.1212/wnl.17.5.427

[ref24] Hughes TA, Ross HF, Mindham RH, Spokes EG (2004). Mortality in Parkinson's disease and its association with dementia and depression. Acta Neurologica Scandinavica 110, 118–123. 15242420 10.1111/j.1600-0404.2004.00292.x

[ref25] Hurt CS, Burn DJ, Hindle J, Samuel M, Wilson K, Brown RG (2013). Thinking positively about chronic illness: an exploration of optimism, illness perceptions and well-being in patients with Parkinson's disease. British Journal of Health Psychology 19, 363–379. 23510498 10.1111/bjhp.12043

[ref26] Hurt CS, Landau S, Burn DJ, Hindle JV, Samuel M, Wilson K, Brown RG (2012). Cognition, coping, and outcome in Parkinson's disease. International Psychogeriatrics 24, 1656–1663. 22612910 10.1017/S1041610212000749

[ref27] Hurt CS, Thomas BA, Burn DJ, Hindle JV, Landau S, Samuel M, Wilson KC, Brown RG (2011). Coping in Parkinson's disease: an examination of the coping inventory for stressful situations. International Journal of Geriatric Psychiatry 26, 1030–1037. 20872797 10.1002/gps.2634

[ref28] Jankovic J, McDermott M, Carter J, Gauthier S, Goetz C, Golbe L, Huber S, Koller W, Olanow C, Shoulson I (1990). Variable expression of Parkinson's disease: a base-line analysis of the DATATOP cohort. The Parkinson Study Group. Neurology 40, 1529–1534. 2215943 10.1212/wnl.40.10.1529

[ref29] Lamers F, Rhebergen D, Merikangas KR, de JP, Beekman AT, Penninx BW (2012). Stability and transitions of depressive subtypes over a 2-year follow-up. Psychological Medicine 42, 2083–2093. 22340131 10.1017/S0033291712000141 PMC11503658

[ref30] Lewis SJG, Foltynie T, Blackwell AD, Robbins TW, Owen AM, Barker RA (2005). Heterogeneity of Parkinson's disease in the early clinical stages using a data driven approach. Journal of Neurology Neurosurgery and Psychiatry 76, 343–348. 10.1136/jnnp.2003.033530 PMC1739569 15716523

[ref31] Menza M, Dobkin RD, Marin H, Mark MH, Gara M, Buyske S, Bienfait K, Dicke A (2009). A controlled trial of antidepressants in patients with Parkinson disease and depression. Neurology 72, 886–892. 19092112 10.1212/01.wnl.0000336340.89821.b3 PMC2677475

[ref32] Menza MA, Robertson HD, Bonapace AS (1993). Parkinson's disease and anxiety: comorbidity with depression. Biological Psychiatry 34, 465–470. 8268331 10.1016/0006-3223(93)90237-8

[ref33] Michl LC, McLaughlin KA, Shepherd K, Nolen-Hoeksema S (2013). Rumination as a mechanism linking stressful life events to symptoms of depression and anxiety: longitudinal evidence in early adolescents and adults. Journal of Abnormal Psychology 122, 339–352. 23713497 10.1037/a0031994 PMC4116082

[ref34] Mioshi E, Dawson K, Mitchell J, Arnold R, Hodges JR (2006). The Addenbrooke's Cognitive Examination Revised (ACE-R): a brief cognitive test battery for dementia screening. International Journal of Geriatric Psychiatry 21, 1078–1085. 16977673 10.1002/gps.1610

[ref35] Moonen AJ, Wijers A, Leentjens AF, Christine CW, Factor SA, Juncos J, Lyness JM, Marsh L, Panisset M, Pfeiffer R, Rottenberg D, Serrano RC, Shulman L, Singer C, Slevin J, McDonald W, Auinger P, Richard IH (2014). Severity of depression and anxiety are predictors of response to antidepressant treatment in Parkinson's disease. Parkinsonism and Related Disorders 20, 644–646. 24679737 10.1016/j.parkreldis.2014.02.025

[ref36] Muthen LK, Muthen BO (2007). Mplus User's Guide. Muthen and Muthen: Los Angeles, CA.

[ref37] Negre-Pages L, Grandjean H, Lapeyre-Mestre M, Montastruc JL, Fourrier A, Lepine JP, Rascol O (2010). Anxious and depressive symptoms in Parkinson's disease: the French cross-sectionnal DoPaMiP study. Movement Disorders 25, 157–166. 19950403 10.1002/mds.22760

[ref38] Nuti A, Ceravolo R, Piccinni A, Dell'Agnello G, Bellini G, Gambaccini G, Rossi C, Logi C, Dell'Osso L, Bonuccelli U (2004). Psychiatric comorbidity in a population of Parkinson's disease patients. European Journal of Neurology 11, 315–320. 15142224 10.1111/j.1468-1331.2004.00781.x

[ref39] Office for National Statistics (2000). Standard Occupational Classification 2000. The Stationery Office: London.

[ref40] Penninx BW, Nolen WA, Lamers F, Zitman FG, Smit JH, Spinhoven P, Cuijpers P, de Jong PJ, van Marwijk HW, van der Meer K, Verhaak P, Laurant MG, de GR, Hoogendijk WJ, van der Wee N, Ormel J, van DR, Beekman AT (2011). Two-year course of depressive and anxiety disorders: results from the Netherlands Study of Depression and Anxiety (NESDA). Journal of Affective Disorders 133, 76–85. 21496929 10.1016/j.jad.2011.03.027

[ref59] Prentice R, Gloeckler L (1978). Regression analysis of grouped survival data with application to breast cancer data. Biometrics 34, 57–67. 630037

[ref41] Qureshi SU, Amspoker AB, Calleo JS, Kunik ME, Marsh L (2012). Anxiety disorders, physical illnesses, and health care utilization in older male veterans with Parkinson disease and comorbid depression. Journal of Geriatric Psychiatry and Neurology 25, 233–239. 23197499 10.1177/0891988712466458

[ref42] Reijnders JSAM, Ehrt U, Weber WEJ, Aarsland D, Leentjens AFG (2008). A systematic review of prevalence studies of depression in Parkinson's disease. Movement Disorders 23, 183–189. 17987654 10.1002/mds.21803

[ref43] Richard IH, McDermott MP, Kurlan R, Lyness JM, Como PG, Pearson N, Factor SA, Juncos J, Serrano RC, Brodsky M, Manning C, Marsh L, Shulman L, Fernandez HH, Black KJ, Panisset M, Christine CW, Jiang W, Singer C, Horn S, Pfeiffer R, Rottenberg D, Slevin J, Elmer L, Press D, Hyson HC, McDonald W (2012). A randomized, double-blind, placebo-controlled trial of antidepressants in Parkinson disease. Neurology 78, 1229–1236. 22496199 10.1212/WNL.0b013e3182516244 PMC3324323

[ref44] Riedel O, Dodel R, Deuschl G, Klotsche J, Forstl H, Heuser I, Oertel W, Reichmann H, Riederer P, Trenkwalder C, Wittchen HU (2012). Depression and care-dependency in Parkinson's disease: results from a nationwide study of 1449 outpatients. Parkinsonism and Related Disorders 18, 598–601. 22118899 10.1016/j.parkreldis.2011.11.007

[ref45] Schrag A, Jahanshahi M, Quinn N (2000). What contributes to quality of life in patients with Parkinson's disease? J. Neurol. Neurosurg. Psychiatry 69, 308–312. 10945804 10.1136/jnnp.69.3.308 PMC1737100

[ref46] Schwab RS, England AC (1969). Projection technique for evaluating surgery for Parkinson's disease In Third Symposium on Parkinson's Disease (ed. F. J. Gillingham and M. C. Donaldson ), pp. 152–157. E&S Livingstone: Edinburgh.

[ref47] Seppi K, Weintraub D, Coelho M, Perez-Lloret S, Fox SH, Katzenschlager R, Hametner EM, Poewe W, Rascol O, Goetz CG, Sampaio C (2011). The movement disorder society evidence-based medicine review update: treatments for the non-motor symptoms of Parkinson's disease. Movement Disorders 26 (Suppl. 3), S42–S80. 22021174 10.1002/mds.23884 PMC4020145

[ref48] Shulman LM, Taback RL, Rabinstein AA, Weiner WJ (2002). Non-recognition of depression and other non-motor symptoms in Parkinson's disease. Parkinsonism and Related Disorders 8, 193–197. 12039431 10.1016/s1353-8020(01)00015-3

[ref49] Silverman MN, Deuster PA (2014). Biological mechanisms underlying the role of physical fitness in health and resilience. Interface Focus 4, 20140040. 25285199 10.1098/rsfs.2014.0040 PMC4142018

[ref50] Starkstein SE, Mayberg HS, Leiguarda R, Preziosi TJ, Robinson RG (1992). A prospective longitudinal study of depression, cognitive decline, and physical impairments in patients with Parkinson's disease. Journal of Neurology Neurosurgery and Psychiatry 55, 377–382. 10.1136/jnnp.55.5.377 PMC489079 1602311

[ref51] StataCorp LP (2009). Stata 11 Base Reference Manual. Stata Press: College Station, TX.

[ref52] Tandberg E, Larsen JP, Aarsland D, Cummings JL (1996). The occurrence of depression in Parkinson's disease. A community-based study. Archives of Neurology 53, 175–179. 8639068 10.1001/archneur.1996.00550020087019

[ref53] Tomlinson CL, Stowe R, Patel S, Rick C, Gray R, Clarke CE (2010). Systematic review of levodopa dose equivalency reporting in Parkinson's disease. Movement Disorders 25, 2649–2653. 21069833 10.1002/mds.23429

[ref54] Vu TC, Nutt JG, Holford NH (2012). Progression of motor and nonmotor features of Parkinson's disease and their response to treatment. British Journal of Clinical Pharmacology 74, 267–283. 22283961 10.1111/j.1365-2125.2012.04192.x PMC3630747

[ref55] Weintraub D, Moberg PJ, Duda JE, Katz IR, Stern MB (2003). Recognition and treatment of depression in Parkinson's disease. Journal of Geriatric Psychiatry and Neurology 16, 178–183. 12967062 10.1177/0891988703256053

[ref56] Weintraub D, Morales KH, Moberg PJ, Bilker WB, Balderston C, Duda JE, Katz IR, Stern MB (2005). Antidepressant studies in Parkinson's disease: a review and meta-analysis. Movement Disorders 20, 1161–1169. 15954137 10.1002/mds.20555 PMC1989731

[ref57] Whitelaw NA, Liang J (1991). The structure of the OARS physical health measures. Medical Care 29, 332–347. 2020202 10.1097/00005650-199104000-00003

[ref58] Zigmond AS, Snaith RP (1983). The Hospital Anxiety and Depression Scale. Acta Psychiatrica Scandinavica 67, 361–370. 6880820 10.1111/j.1600-0447.1983.tb09716.x

