# Supplementary material for: Anxiety and anxious-depression in Parkinson's disease over a 4-year period: a latent transition analysis
Source: Psychol Med. 2015 Nov 23;46(3):657–67. doi: 10.1017/S0033291715002196 (PMC4697304; doi:10.1017/S0033291715002196)
Supplement: Supplementary file 1 [file S0033291715002196sup001.zip › PSM-D-15-00273 Supplementary Figure 1.docx]

**Supplementary Figure S1**

Flow chart showing numbers at each assessment and reasons for loss to follow-up

Consented

N=525

Completed Year 1 assessment

N=513

Completed Year 2 assessment

N=458

Completed Year 3 assessment

N=395

Completed Year 4 assessment

N=329

Withdrew before completing
baseline assessment (N=12)

Lost to follow-up N=55

- Died (N=10)

- Not assessable /
 withdrew due to ill health (N=13)

- Withdrew (other) (N=20)

- Lost contact / moved away (N=9)

- Change of diagnosis (N=3)

Loss to follow-up N=63

- Died (N=19)

- Not assessable /
 withdrew due to ill health (N=13)

- Withdrew (other) (N=12)

- Lost contact / moved away (N=14)

- Change of diagnosis (N=5)

Loss to follow-up N=66
- Died (N=15)

- Not assessable /
 withdrew due to ill health (N=19)

- Withdrew (other) (N=16)

- Lost contact / moved away (N=14)

- Change of diagnosis (N=2)
