# Supplementary material for: Anxiety and anxious-depression in Parkinson's disease over a 4-year period: a latent transition analysis
Source: Psychol Med. 2015 Nov 23;46(3):657–67. doi: 10.1017/S0033291715002196 (PMC4697304; doi:10.1017/S0033291715002196)
Supplement: Supplementary file 1 [file S0033291715002196sup001.zip › PSM-D-15-00273 Supplementary Table 1.docx]

**Supplementary Table S1** Observed demographic and clinical characteristics of cohort at each assessment time-point

| **Variable** | **Year 1** (N=513)  Mean (SD) / Median(range)* / % | **Year 2** (N=458)  Mean (SD) / Median(range)* / % | **Year 3** (N=395)  Mean (SD) / Median(range)* / % | **Year 4** (N=329)  Mean (SD) / Median(range)* / % |
| --- | --- | --- | --- | --- |
| Gender (% Male) | 65.1% | 63.5% | 65.4% | 64.2% |
| Age (Years) | 67.9 (10.3) | 68.4 (10.3) | 69.0 (10.0) | 69.2 (10.0) |
| Age at PD onset (Years) | 61.0 (12.1) | 61.5 (12.1) | 60.3 (11.8) | 59.9 (11.5) |
| UPDRS-III (Total score) | 26.4 (12.0) | 31.0 (13.1) | 32.8 (13.3) | 34.7 (13.5) |
| LEDD (mg/day) | 600 (0-7565)* | 645 (0-8695)* | 731 (0-3212)* | 800 (0-4105)* |
| Antidepressant/anxiolytic use (% yes) | 23.5% | 21.9% | 28.7% | 30.4% |
| Hoehn and Yahr (% stage I/II-III/IV-V) | 12.6/80.2/7.2% | 9.6/79.9/10.5% | 7.1/82.8/10.1% | 3.0/72.6/17.3% |
| MMSE (Total score) | 27.9 (2.5) | 27.8 (2.8) | 27.9 (2.7) | 27.8 (2.8) |
| ACE-R (Total score) | 86.4 (10.7) | 86.3 (10.8) | 87.3 (10.9) | 87.6 (11.2) |
| ACE-R ≤83 (%) | 29.7% | 32.2% | 25.6% | 25.5% |
| HADS-Depression | 6.3 (3.7) | 6.5 (3.9) | 6.5 (3.7) | 6.7 (3.9) |
| HADS-Depression ≥11 (%) | 13.0% | 14.9% | 14.5% | 14.1% |
| HADS-Anxiety | 7.2 (4.5) | 7.4 (4.3) | 7.1 (4.2) | 7.2 (4.4) |
| HADS-Anxiety ≥11 (%) | 22.0% | 25.0% | 20.1% | 25.3% |
| HAM-D (Total) | 5.7 (5.3) | 6.0 (5.2) | 6.7 (5.1) | 6.6 (4.9) |
| HAM-D≥10/16 (%) | 20.5/5.6% | 20.5/5.4% | 23.6/7.5% | 24.1/5.7% |

**Abbreviations:** UPDRS, Unified Parkinson’s Disease rating Scale; LEDD, Levodopa equivalent daily dose; ACE-R, Addenbrooke’s Cognitive Examination (Revised); MMSE, Mini-Mental State Examination; HADS, Hospital Anxiety and Depression Scale; HAM-D, Hamilton Depression Rating Scale.
