# Supplementary material for: Anxiety and anxious-depression in Parkinson's disease over a 4-year period: a latent transition analysis
Source: Psychol Med. 2015 Nov 23;46(3):657–67. doi: 10.1017/S0033291715002196 (PMC4697304; doi:10.1017/S0033291715002196)
Supplement: Supplementary file 1 [file S0033291715002196sup001.zip › PSM-D-15-00273 Supplementary Table 2.docx]

**Supplementary Table S2** Proportion (%) patients of patients in the latent classes receiving antidepressant/anxiolytic medication over the four years

|  | **Year 1**  (N=513) | **Year 2**  (N=458) | **Year 3**  (N=395) | **Year 4**  (N=329) |
| --- | --- | --- | --- | --- |
| High anxiety + depression | 54.2% | 36.8% | 57.9% | 42.9% |
| Moderate anxiety + depression | 40.0% | 37.0% | 42.6% | 33.3% |
| Moderate anxiety | 34.4% | 26.7% | 34.8% | 42.0% |
| Psychologically healthy | 13.7% | 14.5% | 20.0% | 23.5% |
| (Whole sample) | (23.5%) | (21.9%) | (28.7%) | (30.4%) |
