# Supplementary material for: Anxiety and anxious-depression in Parkinson's disease over a 4-year period: a latent transition analysis
Source: Psychol Med. 2015 Nov 23;46(3):657–67. doi: 10.1017/S0033291715002196 (PMC4697304; doi:10.1017/S0033291715002196)
Supplement: Supplementary file 1 [file S0033291715002196sup001.zip › PSM-D-15-00273 Supplementary Table 3.docx]

**Supplementary Table S3** Estimated transition frequencies by year.

| **Subtype** | **High anxiety + depression** | **Moderate anxiety + depression** | **Moderate anxiety** | **Psychologically healthy** |
| --- | --- | --- | --- | --- |
| **Year 1** | **Transitions in year 2** | | | |
| **High anxiety + depression** | 73.8% | 0% | 26.2% | 0% |
| **Moderate anxiety + depression** | 11.6% | 62.9% | 9.9% | 15.5% |
| **Moderate anxiety** | 16.4% | 9.3% | 69.2% | 5.2% |
| **Psychologically healthy** | 0% | 3.6% | 8.9% | 87.5% |
| **Year 2** | **Transitions in year 3** | | | |
| **High anxiety + depression** | 42.4% | 22.6% | 30.5% | 4.6% |
| **Moderate anxiety + depression** | 0% | 81.0% | 19.0% | 0% |
| **Moderate anxiety** | 4.0% | 13.9% | 58.7% | 23.4% |
| **Psychologically healthy** | 0% | 3.2% | 2.8% | 93.9% |
| **Year 3** | **Transitions in year 4** | | | |
| **High anxiety + depression** | 52.4% | 24.0% | 14.3% | 9.2% |
| **Moderate anxiety + depression** | 24.1% | 62.5% | 7.9% | 5.5% |
| **Moderate anxiety** | 1.4% | 3.9% | 88.1% | 6.7% |
| **Psychologically healthy** | 0% | 1.1% | 1.9% | 96.9% |

**Note:**

Shaded cells represent cases remaining in same LTA class on successive years
